# Induction of β-Lactamase Activity and Decreased β-Lactam Susceptibility by CO_2_ in Clinical Bacterial Isolates

**DOI:** 10.1128/mSphere.00266-17

**Published:** 2017-07-19

**Authors:** Nathan Mullen, Hugo Raposo, Polyxeni Gudis, Linsey Barker, Romney M. Humphries, Bryan H. Schmitt, Ryan F. Relich, Meghan May

**Affiliations:** a Department of Biomedical Sciences, University of New England College of Osteopathic Medicine, Biddeford, Maine, USA; b Department of Pathology and Laboratory Medicine, David Geffen School of Medicine at UCLA, Los Angeles, California, USA; c Department of Pathology and Laboratory Medicine, Indiana University School of Medicine, Indianapolis, Indiana, USA; JMI Laboratories

**Keywords:** BLA, *Francisella*, *Haemophilus*, SHV, TEM, antimicrobial resistance, β-lactamases, carbon dioxide

## Abstract

Antimicrobial susceptibility testing of clinical isolates is a crucial step toward appropriate treatment of infectious diseases. The clinical isolate *Francisella philomiragia* 14IUHPL001, recently isolated from a 63-year-old woman with atypical pneumonia, featured decreased susceptibility to β-lactam antibiotics when cultivated in 5% CO_2_. Quantitative β-lactamase assays demonstrated a significant (*P* < 0.0001) increase in enzymatic activity between bacteria cultivated in 5% CO_2_ over those incubated in ambient air. The presence of β-lactamase genes *bla*_TEM_ and *bla*_SHV_ was detected in the clinical isolate *F. philomiragia* 14IUHPL001 by PCR, and the genes were positively identified by nucleotide sequencing. Expression of *bla*_TEM_ and *bla*_SHV_ was detected by reverse transcription-PCR during growth at 5% CO_2_ but not during growth in ambient air. A statistically significant alkaline shift was observed following cultivation of *F. philomiragia* 14IUHPL001 in both ambient air and 5% CO_2_, allowing desegregation of the previously reported effects of acidic pH from the currently reported effect of 5% CO_2_ on *bla*_TEM_ and *bla*_SHV_ β-lactamases. To ensure that the observed phenomenon was not unique to *F. philomiragia*, we evaluated a clinical isolate of *bla*_TEM_-carrying *Haemophilus influenzae* and found parallel induction of *bla*_TEM_ gene expression and β-lactamase activity at 5% CO_2_ relative to ambient air.

**IMPORTANCE** β-Lactamase induction and concurrent β-lactam resistance in respiratory tract pathogens as a consequence of growth in a physiologically relevant level of CO_2_ are of clinical significance, particularly given the ubiquity of TEM and SHV β-lactamase genes in diverse bacterial pathogens. This is the first report of β-lactamase induction by 5% CO_2_.

## Abstract (precis)

β-Lactamase induction and concurrent β-lactam resistance in respiratory tract pathogens as a consequence of growth in a physiologically relevant level of CO_2_ are of clinical significance, particularly given the ubiquity of TEM and SHV β-lactamase genes in diverse bacterial pathogens. This is the first report of β-lactamase induction by 5% CO_2_.

## INTRODUCTION

Members of the genus *Francisella* are aerobic Gram-negative coccobacilli, and many species are known to be professional pathogens or opportunists of many animal species (1–4). *Francisella philomiragia* is an uncommon pathogen of humans, having primarily been reported in near-drowning victims and individuals with chronic granulomatous disease (3). *F. philomiragia* typically infects the lower respiratory tract but has also been reported to cause septicemia and meningitis (3). A recent case involving a 63-year-old woman who presented with shortness of breath, nonproductive cough, and bilateral peripheral edema yielded the isolate *F. philomiragia* strain 14IUHPL001 (1). Antimicrobial susceptibility testing of this isolate was conducted in ambient air and 5% CO_2_ atmospheres. Interestingly, this bacterium became less susceptible to β-lactam antibiotics when incubated in an atmosphere enriched with 5% CO_2_.

Known mechanisms of reduced susceptibility to β-lactams include lack of a cell wall, alterations in penicillin-binding proteins, or production of β-lactamase (5). β-Lactamases hydrolyze the β-lactam ring that is present in all β-lactam antibiotics and rapidly degrade the molecule (6). A small number of extended-spectrum β-lactamases, including TEM-1, TEM-6, TEM-10, and SHV, have been suggested previously to be regulated by CO_2_ or pH (7–9). These enzymes’ genes are typically found on plasmids but can also be integrated into the bacterial genome. In order to determine the mechanism of CO_2_-derived susceptibility changes in *F. philomiragia* 14IUHPL001, we sought to characterize β-lactamase activity phenotypically, genotypically, and transcriptionally in ambient air and 5% CO_2_.

## RESULTS

### Antimicrobial susceptibility.

*F. philomiragia* strain 14IUHPL001 generally became less susceptible to β-lactams when incubated in 5% CO_2_ (Table 1) *F. philomiragia* FSC144^T^ was 8-fold more susceptible to cefepime when incubated in ambient air than CO_2_. *F. philomiragia* 14IUHPL001, most notably, was 64-fold more susceptible to oxacillin (Table 1). In both cases, the susceptibility of *F. philomiragia* strains 14IUHPL001 and FSC144^T^ demonstrated a trend toward β-lactam resistance when incubated with 5% CO_2_, which warranted further investigation.

**TABLE 1:** Antimicrobial susceptibility of *Francisella philomiragia* under different atmospheric conditions

Antimicrobial agent(s)	MIC (μg/ml) for:			
	14IUHPL001		FSC144^T^	
	Ambient air	CO_2_	Ambient air	CO_2_
Amikacin	≤0.5	≤0.5	≤0.5	≤0.5
Amoxicillin-clavulanic acid	1	16	1	4
Ampicillin	32	>32	32	>32
Aztreonam	4	32	2	8
Cefepime	4	32	1	8
Ceftazidime	≤0.5	≤0.5	≤0.5	≤0.5
Ceftriaxone	≤0.5	≤0.5	≤0.5	≤0.5
Cefazolin	2	>32	16	>32
Ciprofloxacin	≤0.25	≤0.25	≤0.25	≤0.25
Colistin	>8	>8	>8	>8
Doripenem	≤0.25	1	≤0.25	0.50
Doxycycline	≤1	≤1	≤1	≤1
Ertapenem	≤0.25	1	≤0.25	≤0.25
Gentamicin	≤0.5	≤0.5	≤0.5	≤0.5
Imipenem	≤0.25	0.50	≤0.25	≤0.25
Levofloxacin	≤2	≤2	≤2	≤2
Meropenem	≤0.25	0.5	≤0.25	≤0.25
Moxifloxacin	≤0.25	≤0.25	≤0.25	≤0.25
Oxacillin	≤0.25	>16	>16	>16
Polymyxin B	>4	>4	>4	>4
Ticarcillin-clavulanic acid	≤4	≤4	≤4	≤4
Tigecycline	≤0.25	≤0.25	≤0.25	≤0.25
Tobramycin	≤0.5	≤0.5	≤0.5	≤0.5
Trimethoprim-sulfamethoxazole	>4	>4	>4	>4

### β-Lactamase activity.

**FIG 1:**
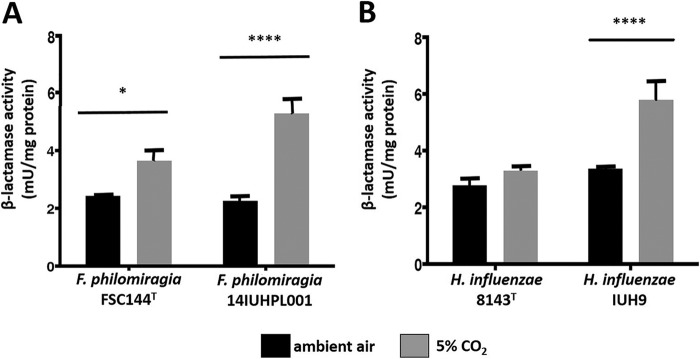
β-Lactamase activity in *F. philomiragia* and *H. influenzae*. Enzymatic units of β-lactamase activity were measured for clinical isolates 14IUHPL0001 and IUH9 and type strains FSC144 and 8143 cultivated in either ambient air (black bars) or 5% CO_2_ (gray bars). Enzymatic units were normalized to milligrams of total bacterial protein. (A) Both *F. philomiragia* strains produced significantly (*, *P* < 0.05; ****, *P* < 0.0001) higher levels of β-lactamase in 5% CO_2_ relative to ambient air, and strain 14IUHPL0001 produced significantly more β-lactamase in 5% CO_2_ relative to FSC144^T^. (B) Strain IUH9 produced significantly higher levels of β-lactamase in 5% CO_2_ relative to growth in ambient air and strain 8143^T^ grown in either 5% CO_2_ or ambient air. There was no significant difference in activities when strain 8143^T^ was grown in 5% CO_2_ or ambient air.

Nitrocefin disk testing confirmed the presence of β-lactamase activity. *F. philomiragia* FSC144^T^ incubated in 5% CO_2_ had a 1.5-fold increase in β-lactamase activity compared to atmospheric air (*P* < 0.01). In comparison, *F. philomiragia* strain 14IUHPL001 incubated with CO_2_ had a 2.4-fold increase in β-lactamase activity compared to atmospheric air (*P* < 0.0001). When comparing both strains of *F. philomiragia* that were incubated with CO_2_, *F. philomiragia* 14IUHPL001 had a 1.5-fold increase in β-lactamase activity over *F. philomiragia* FSC144^T^ (*P* < 0.01). There was no significant difference between *F. philomiragia* FSC144^T^ and 14IUHPL001 incubated in atmospheric air (Fig. 1A). *Haemophilus influenzae* IUH9 incubated in 5% CO_2_ was significantly increased compared to *H. influenzae* IUH9 incubated in ambient air and *H. influenzae* 8143^T^ incubated in 5% CO_2_ (*P* < 0.0001). No significant differences were observed between *H. influenzae* 8143^T^ incubated in ambient air versus 5% CO_2_ or 8143^T^ versus IUH9 incubated in ambient air (Fig. 1B).

### *bla*_TEM_ and *bla*_SHV_ amplification.

**FIG 2:**
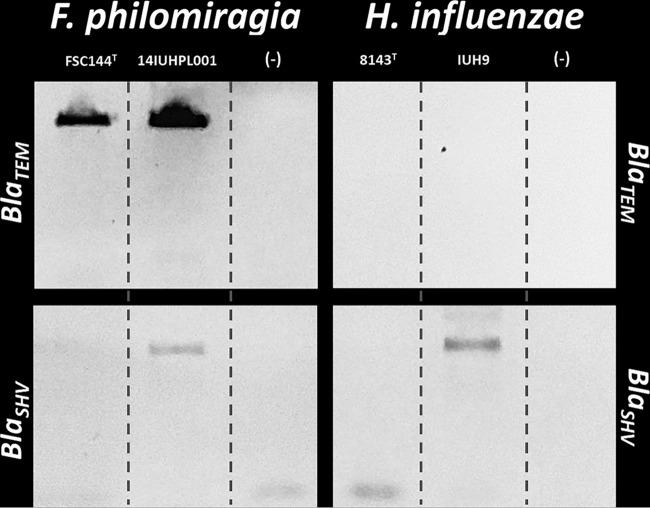
Interrogation of *F. philomiragia* and *H. influenzae* for TEM and SHV family β-lactamase genes. *bla*_TEM_-specific primers yielded PCR amplicons from both *F. philomiragia* 14IUHPL0001 and FSC144^T^, but not from *H. influenzae* IUH9 or 8143^T^ (top panel). *bla*_SHV_-specific primers yielded PCR amplicons from clinical isolates *F. philomiragia* 14IUHPL0001 and *H. influenzae* IUH9 but not from either type strain (bottom panel). Negative controls (−) for all reactions were template-free reaction mixtures containing all reagents.

Primers designed to amplify *bla*_TEM_ generated the predicted product of 850 bp in *F. philomiragia* 14IUHPL001 and in the type strain *F. philomiragia* FSC144 but failed to detect the gene in either strain of *H. influenzae* (Fig. 2). Primers designed to amplify *bla*_SHV_ generated the predicted 768-bp product in *F. philomiragia* 14IUHPL001 and *H. influenzae* IUH9 but not in either type strain (Fig. 2). *F. philomiragia* 14IUHPL001 and FSC144^T^ produced *bla*_TEM_ transcript in 5% CO_2_ but not in ambient air. Similarly, *F. philomiragia* 14IUHPL001 and *H. influenzae* IUH9 produced *bla*_SHV_ transcript in 5% CO_2_ but not in ambient air. As expected, no *bla*_TEM_ transcript was detected in *H. influenzae* IUH9 or 8143^T^, and no *bla*_SHV_ transcript was detected in *H. influenzae* 8143^T^ or *F. philomiragia* FSC144^T^, regardless of atmospheric conditions (Table 2).

**TABLE 2:** Expression of *bla*_TEM_ and *bla*_SHV_ under different atmospheric conditions

Gene and condition	Expression of gene under condition shown^a^			
	*F. philomiragia*		*H. influenzae*	
	14IUHPL0001	FSC144T	IUH9	8143T
5% CO_2_				
* bla*_TEM_	+	+	−	−
* bla*_SHV_	+	−	+	−
Ambient air				
* bla*_TEM_	−	−	−	−
* bla*_SHV_	−	−	−	−

a The symbols “+” and “−” indicate the presence or absence, respectively, of RNA/cDNA amplification by reverse transcription-PCR.

### Nucleotide sequencing and phylogenetic analysis.

**FIG 3:**
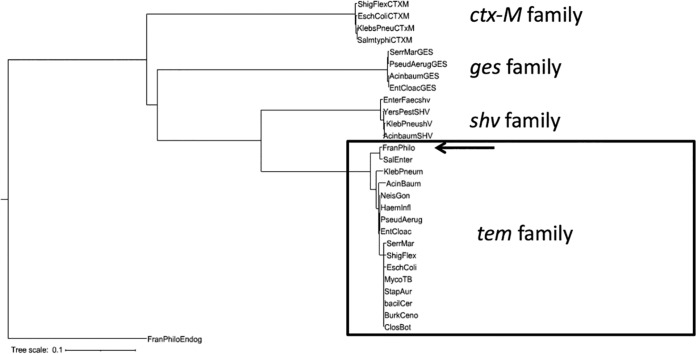
Phylogenetic analysis of *bla*_TEM_. Phylogenetic analysis performed on the translation of the sequenced amplicon indicated that the 14IUHPL0001 and type strain FSC144^T^ allele clusters with the TEM family β-lactamases. The endogenous chromosomal β-lactamase gene from the type strain FSC144^T^ served as the outgroup.

Sequencing of the *F. philomiragia*-derived *bla*_TEM_ amplicons indicated that strains 14IUHPL001 and FSC144^T^ are carrying an identical copy of the gene. The derived sequence identified it as a member of the TEM family of β-lactamase genes (GenBank accession no. KT781076). Sequencing of the 768-bp *bla*_SHV_ product from both *F. philomiragia* 14IUHPL001 and *H. influenzae* IUH9 showed 100% identity with that of *Klebsiella pneumoniae* KPNIH27. While the *F. philomiragia*-derived *bla*_TEM_ had a unique nucleotide sequence, phylogenetic analysis indicates that it clearly still belongs to the TEM family of β-lactamase genes (Fig. 3).

### pH measurements.

**FIG 4:**
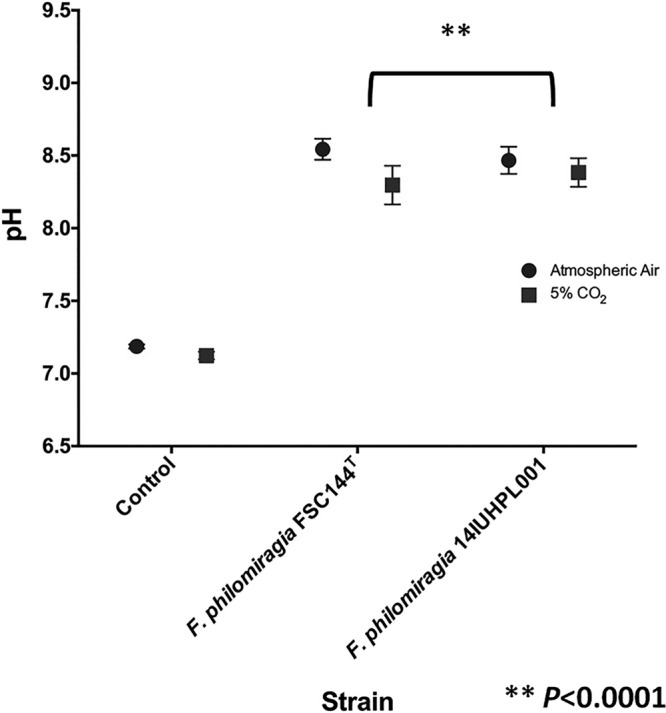
Cultivation of *F. philomiragia* in 5% CO_2_ or ambient air generates an alkaline pH shift. Both 14IUHPL0001 and FSC144^T^ significantly shifted the growth medium pH higher in either ambient air (circles) or 5% CO_2_ (squares) relative to incubated, uninoculated medium.

Following incubation, a significant alkaline shift in pH was observed for both *F. philomiragia* 14IUHPL001 and the type strain, FSC144, when incubated in CO_2_ or ambient air (Fig. 4) (*P* < 0.0001). Differences in pH between the CO_2_ and atmospheric air incubation methods were not significant for *F. philomiragia* FSC144^T^ and *F. philomiragia* 14IUHPL001. *In situ* measurements of pH indicated that rapid changes did not occur. Our analysis of this phenomenon with *F. philomiragia* naturally decouples the effects of atmospheric CO_2_ and acidic pH on *bla*_TEM_ activity levels and thus β-lactam susceptibility.

## DISCUSSION

Changes in susceptibility to β-lactam antibiotics were observed for *F. philomiragia* strain 14IUHPL001 when grown under different atmospheric conditions. Growth in 5% CO_2_ resulted in significant (*P* < 0.05) deviations from the MIC in ambient air of β-lactam antibiotics (Table 1). There are no established interpretative criteria for any of the antimicrobials tested versus *F. philomiragia*, limiting our ability to discuss clinically relevant breakpoints; however, the magnitude of susceptibility decrease is substantial and in all likelihood meaningful during disease. The MICs of other antimicrobial classes did not change regardless of incubation conditions, indicating a CO_2_-inducible change uniquely impacting β-lactam–*F. philomiragia* interactions.

The presence of β-lactamase was qualitatively detected in both *F. philomiragia* strain 14IUHPL001 and the type strain, FSC144, grown in either ambient air or 5% CO_2_ with nitrocefin disks. Quantitative, colorimetric β-lactamase activity assays for both strains of *F. philomiragia* resulted in a significant increase in β-lactamase activity level when incubated in 5% CO_2_. *F. philomiragia* strain 14IUHPL001 had a 2.4-fold increase in β-lactamase activity when incubated in 5% CO_2_ compared to atmospheric air (*P* < 0.0001). The type strain, FSC144, also had increased β-lactamase activity in 5% CO_2_, but the effect was less striking (1.5-fold change; *P* < 0.05) (Fig. 1A). These findings are consistent with the decrease in β-lactam susceptibility that was observed for both strains of *F. philomiragia* in the presence of 5% CO_2_ (Table 1) and indicate that the organisms are expressing β-lactamase genes that are inducible by CO_2_. Previous reports have described changes in penicillin and piperacillin susceptibility of *Escherichia coli* isolates carrying the β-lactamases TEM-1, TEM-2, and SHV at elevated CO_2_ levels or acidic pH (7). Examination of *bla*_TEM_ family sequences in public databases indicates a very high level of nucleotide identity across diverse Gram-negative and Gram-positive species, indicating that it is horizontally transferred and strictly conserved. Identity searches using the BLAST algorithm (10) further confirmed that the nomenclature of many TEM family β-lactamases is highly redundant (>98% identity across all TEM identifiers), and the TEM identifiers 1 and 2 (100% identity) are not indicative of distinct alleles. However, point mutations within *bla*_TEM_ have been used epidemiologically to track both outbreak strains and horizontal transfer to new species (11). Regardless of the specific allele, phylogenetic analysis clearly indicates that one of the β-lactamase genes detected in *F. philomiragia* 14IUHPL001 belongs to the *bla*_TEM_ family (Fig. 3).

The presence of a *bla*_TEM_ β-lactamase gene in *F. philomiragia* 14IUHPL001 was confirmed by PCR with *bla*_TEM_-specific primers and sequencing of the resulting product (Fig. 2 [GenBank accession no. KT781076]). The same gene was amplified from the type strain of *F. philomiragia*. While *bla*_TEM_ was not annotated in the FSC144^T^ genome (12), the gene was detected from extracted plasmid DNA and therefore not represented on the chromosome. Unrelated chromosomal β-lactamase genes are annotated, however. Although *F. philomiragia* is reported to carry a cryptic plasmid, pFPHIo1, no β-lactamase genes are carried on this plasmid (12). Although *bla*_TEM_ expression likely explains increases in β-lactamase activity in *F. philomiragia* 14IUHPL001 and FSC144^T^ relative to growth in ambient air, it fails to explain the significant difference in β-lactamase activities between the two strains at 5% CO_2_ (Fig. 1A). We then interrogated 14IUHPL001 and FSC144 for *bla*_SHV_, another β-lactamase gene that has been reported to be affected by CO_2_ (7–9). The *bla*_SHV_ gene was detected in 14IUHPL001 but not FSC144^T^ (Fig. 2). The presence of an additional CO_2_-regulated β-lactamase gene is likely responsible for the significant difference in activities found.

To ensure that the observed effect was not unique to *F. philomiragia*, quantitative β-lactamase activity was measured for the *bla*_SHV_-bearing *H. influenzae* strain IUH9 as well. As observed for *F. philomiragia*, *H. influenzae* IUH9 produced significantly more β-lactamase activity in 5% CO_2_ relative to (i) growth in ambient air and (ii) the *bla*_SHV_-deficient type strain, 8143, under either atmospheric condition. Taken together, these data indicate that at least *bla*_SHV_ is associated with elevated β-lactamase activity at 5% CO_2_ regardless of the bacterial species producing the protein.

Given the promiscuity of TEM and SHV β-lactamases and the reported differential β-lactam susceptibility of *Escherichia coli* strains harboring TEM β-lactamases in elevated CO_2_, *F. philomiragia* strain 14IUHPL001 was positively interrogated for *bla*_TEM_. Livermore and Corkill also reported decreased β-lactam susceptibility in acidic pH (9). In order to distinguish the effects of atmospheric CO_2_ from drops in pH during incubation, we measured the change in pH (ΔpH) generated by 14IUHPL001 and FSC144^T^ following incubation in either ambient air or 5% CO_2_. Both 14IUHPL001 and FSC144^T^ generated a net positive ΔpH (alkaline shift) relative to uninoculated, contemporarily incubated media in both atmospheric conditions. There was no significant difference in pH values between uninoculated growth media incubated in 5% CO_2_ and atmospheric air (Fig. 4). β-Lactamase induction at 5% CO_2_ in the absence of an acid shift is a critical finding given the inherent clinical significance of this activity in a respiratory pathogen as a consequence of growth in 5% CO_2_ directly. The atmospheric conditions at the alveolar surface are not directly comparable to ambient air (partial CO_2_ pressure [pCO_2_] of 0). When the atmospheric gases equilibrate with the blood in the alveoli, the alveolar pCO_2_ elevates to 40 mm Hg, or approximately 5.3%. If induction of *bla*_TEM_ was secondary to acid production during laboratory incubation, the clinical relevance of this finding would still remain only partially defined. Our findings indicate a direct effect of a physiologically relevant level of atmospheric CO_2_ independent of acidic pH on the *bla*_TEM_-derived β-lactamase activity level, and thus β-lactam susceptibility during infection. Antimicrobial susceptibility testing (AST) performed under standard conditions (i.e., ambient air) would indicate that bacterial pathogens carrying *bla*_TEM_ are susceptible to β-lactam treatment. If such isolates are isolated from the lower respiratory tract, β-lactam treatment failures that would not be consistent with the reported AST results are predictable.

Antimicrobial susceptibility testing is essential for appropriate treatment decisions during bacterial infection. Previous reports have questioned the clinical significance of changes induced by CO_2_ on β-lactamase activity (13). Our results clearly demonstrate the clinical relevance of CO_2_ regulation of β-lactamase during lower respiratory tract infections. TEM and SHV family β-lactamases have been detected in numerous pathogens associated with infection of the lung, including *Acinetobacter baumannii*, *Burkholderia cepacia*, *Klebsiella pneumoniae*, *Pseudomonas aeruginosa*, *Staphylococcus aureus*, *Stenotrophomonas maltophilia*, and *Yersinia pestis*. Our findings therefore suggest that standard best practices for antimicrobial susceptibility testing could include physiologically relevant conditions in the future.

## MATERIALS AND METHODS

### Strains and culture conditions.

*Francisella philomiragia* strains FSC144^T^ (ATCC 25015) (4, 14) and 14IUHPL001 (1) were cultured on chocolate agar (Remel, Lenexa, KS) at 37°C in either ambient air or 5% CO_2_. Bacteria were harvested from plates and resuspended in 1× phosphate-buffered saline (PBS). Following one PBS wash, the bacteria were pelleted, and the mass of each pellet was measured using an AG285 balance (Mettler Toledo, Columbus, OH). *Haemophilus influenzae* strains 8143 (ATCC 33391) and IUH9 were cultured on chocolate agar in either ambient air or 5% CO_2_. Strain IUH9 was selected from a panel of clinical isolates because growth in ambient air was tolerated and molecular screening indicated that it carried *bla*_TEM_.

### Antimicrobial susceptibility testing.

*F. philomiragia* strains FSC144^T^ and 14IUHPL001 were tested by broth microdilution using panels prepared in house with cation-adjusted Mueller-Hinton broth (CAMHB [Difco, BD Sparks, MD]), according to CLSI standards. Isolates were subcultured twice from frozen stocks on chocolate agar plates, and 5 colonies were picked and suspended in saline to achieve a concentration equivalent to a 0.5 McFarland standard (CLSI M07-A10; January 2015). Panels were inoculated in duplicate; one panel was incubated at 35°C for 24 h in ambient air, and the other panel was incubated at 35°C for 24 h in an atmosphere enriched with 5% CO_2_. Antimicrobial susceptibility testing (AST) in both atmospheres was repeated once to gauge reproducibility.

### pH testing.

Quantitative measurement of growth medium pH postincubation was performed as described by Livermore (9) using a model 125 pH meter (Corning, Corning, NY). Measurements were taken for FSC144^T^, 14IUHPL001, and uninoculated chocolate agar incubated at 37°C after 24 h in ambient air and 5% CO_2_. To assess whether rapid pH shift was occurring when removing growth medium from the 5% CO_2_ incubator, a sterile chocolate agar plate was acclimated in 5% CO_2_ for 3 h. The pH was measured *in situ* and compared to the pH of uninoculated medium in ambient air.

### β-Lactamase activity.

Qualitative assessment of β-lactamase activity was made for *F. philomiragia* FSC144^T^ and 14IUHPL001 using nitrocefin disks according to the manufacturer’s instructions (Remel, San Diego, CA). Quantitative β-lactamase activity was measured using β-lactamase activity colorimetric assay reagents according the manufacturer’s specifications (Bio-Vision, Milpitas, CA). Preweighed bacterial pellets (*F. philomiragia* FSC144^T^ and 14IUHPL001 or *H. influenzae* 8143^T^ or IUH9, grown in ambient air or 5% CO_2_) were suspended in 5 μl of β-lactamase assay buffer per mg of bacteria. Bacterial cell suspensions were then sonicated in an ice bath using a Sonifier cell disruptor 200 (Branson Ultrasonic Corps, Danbury, CT) for 10 s continuously with a 30-s cool down time, for a total of 6 cycles. Samples were then centrifuged at 16,000 × *g* at 4°C for 20 min. The supernatant for each sample was transferred to a new 1.5-ml microcentrifuge tube and stored on ice. To ensure enzymatic activity fell within the linear range of the nitrocefin standard, samples were diluted 2-fold, 5-fold, and 10-fold. Sample blanks consisted of substrate-free assay buffer. The absorbance (λ = 490) was measured kinetically for 45 min at room temperature using a SpectraMax M5 (Molecular Devices, Sunnyvale, CA). β-Lactamase activity was calculated using the equation β-lactamase activity = (*B*/Δ*T* × *V*) × *D*, where *B* (nanomoles) represents the amount of nitrocefin hydrolyzed during the change in time (Δ*T* [minutes]), *V* (milliliter) represents the amount of sample added to the reaction vessel, and *D* represents the dilution factor. Enzymatic activity was normalized to milligrams of bacteria.

### Nucleic acid extraction.

Bacterial DNA was extracted using a QIAprep mini-spin kit (Qiagen, Valencia, CA) following the manufacturer’s protocol specifications. Purified DNA was quantified by measuring absorbance at an optical density of 260 nm (OD_260_) and OD_280_. Bacterial RNA was extracted following cultivation in either ambient air or 5% CO_2_ using TRIzol reagent followed by RNase-free DNase I treatment (Thermo Fisher Scientific, Waltham, MA) according to the manufacturer’s specifications.

### Nucleic acid amplification.

One hundred nanograms of purified DNA was used as the template for each PCR. Amplification of *bla*_TEM_ was performed by initial denaturation at 94°C followed by 45 cycles at 94°C (30 s), 50°C (30 s), and 72°C (70 s) using the following primers: 5′ ATG AGT ATT CAA CAT TTT CGT GTC G 3′ (forward) and 5′ TAC CAA TGC TTA ATC AGT GA 3′ (reverse). Amplification of *bla*_SHV_ was performed by initial denaturation at 94°C followed by 45 cycles at 94°C (30 s), 58°C (30 s), and 72°C (70 s) using the following primers: 5′ TTA ACT CCC TGT TAG CCA 3′ (forward) and 5′ GAT TTG CTG ATT TCG CCC 3′ (reverse) (15). A 5-min final extension was performed at 72°C for each reaction. Products were amplified with GoTaq G2 colorless master mix reagents (Promega, Madison, WI) and purified using the PureLink PCR purification system (Life Technologies, Inc., Carlsbad, CA) according to the manufacturer’s instructions. Positive and negative controls included amplification of a portion of the 16S rRNA gene using universal bacterial primers (19) and reagents without DNA template, respectively. One hundred nanograms of purified RNA was amplified using SuperScript IV reverse transcriptase PCR reagents (Life Technologies, Inc.) according to the manufacturer’s instructions. *F. philomiragia* FSC144^T^ and 14IUHPL001 and *H. influenzae* 8143^T^ and IUH9 RNAs were interrogated for *bla*_TEM_ and *bla*_SHV_ transcript using the aforementioned primer sets as follows: (i) reverse transcription at 55°C for 10 min followed by enzyme inactivation at 80°C for 10 min; (ii) cDNA amplification via 35 cycles at 94°C (30 s), 50°C (30 s), and 72°C (70 s); (iii) a 5-min final extension at 72°C. Positive and negative controls included amplification of a portion of 16S rRNA (19), PCR (reverse transcriptase free) with RNA templates, and reagents without RNA template, respectively.

### Nucleotide sequencing and phylogenetic analysis.

All DNA amplicons were sequenced using four-dye fluorescent dideoxy labeling methods at the University of Florida Interdisciplinary Center for Biotechnology Research. Sequence reads were assembled using Sequencher 4.7 (Gene Codes, Ann Arbor, MI). A phylogenetic tree featuring multiple β-lactamase gene families was generated using Clustal Omega (16) and visualized using iTOL 2.0 (17). Reference sequences were obtained from GenBank (18) with the following accession numbers: KJU60142.1, YP_009062986.1, AJC64567.1, ACZ37308.1, ACH73002.1, ADD96657.1, ABA60617.1, NP_052173.1, YP_006959642.1, CAA38428.1, AAQ73497.1, KJG77969.2, AAB39956.1, ACV20891.1, KIN80010.1, EWD96542.1, AAV38100.1, EDR30442.1, ABN49114.1, ABS12043.1, YP_005351450.1, YP_009061958.1, YP_009090730.1, BAP75641.1, BAO51997.1, ABD75721.1, ADJ94120.1, and EET21660.1.

### Statistical procedures.

The effect of bacterial culture in 5% CO_2_ on β-lactamase activity (*n =* 8 independent replications each) and bacterial culture on pH (*n =* 3 independent replications each) was analyzed by analysis of variance and by Fisher’s protected least significant difference test for *post hoc* comparisons when main effects were significant. Deviations in MIC during growth in 5% CO_2_ relative to the MIC during growth in ambient air were detected by χ^2^ goodness-of-fit analysis. All statistical procedures were performed using Prism v6.0c (GraphPad Software, Inc., La Jolla, CA). *P* values of <0.05 were considered significant.

### Accession number(s).

The sequence of the product of the *F. philomiragia* 14IUHPL001 *bla*_TEM_ β-lactamase gene has been submitted to the GenBank database under accession no. KT781076.
